# Prevalence Survey of Selected Bovine Pathogens in Water Buffaloes in the North Region of Brazil

**DOI:** 10.1155/2014/603484

**Published:** 2014-01-19

**Authors:** Jenevaldo Barbosa da Silva, Priscilla Nunes dos Santos, Gustavo Nunes de Santana Castro, Adivaldo Henrique da Fonseca, José Diomedes Barbosa

**Affiliations:** ^1^Laboratório de Imunoparasitologia, Departamento de Patologia Veterinária, Faculdade de Ciências Agrárias e Veterinárias FCAV-UNESP, Via de Acesso Professor Paulo Donato Castellane s/n, 14884-900 Jaboticabal, SP, Brazil; ^2^Laboratório de Doenças Parasitárias, Departamento de Epidemiologia e Saúde Pública, Universidade Federal Rural de Rio de Janeiro (UFRRJ), BR 465 Km 7, 23890-000 Seropédica, RJ, Brazil; ^3^Instituto de Medicina Veterinária, Universidade Federal do Pará (UFPA), Rodovia BR 316 Km 61, Bairro Saudade, 68740-970 Castanhal, PA, Brazil

## Abstract

Although the largest buffalo herd in the occident is in the north region of Brazil, few studies have been conducted to assess the prevalence of selected parasitic diseases in buffalo herd. The present study was therefore conducted to investigate the epidemiological of *Toxoplasma gondii, Neospora caninum, Anaplasma marginale, Babesia bigemina,* and *Babesia bovis* in water buffaloes in the north region of Brazil. A total of 4796 buffalo blood samples were randomly collected from five provinces and simultaneously analyzed by the IFAT and ELISA. The serological prevalence of *T. gondii* and *N. caninum* was 41.3% and 55.5% in ELISA and 35.7% and 48.8% in IFAT, respectively. The overall prevalence of *A. marginale, B. bovis, and B. bigemina* was 63%, 25%, and 21% by ELISA and 50.0%, 22.5%, and 18.8% by IFAT, respectively. This study shows valuable information regarding the serological survey of selected bovine pathogens in water buffaloes in the north region of Brazil which will likely be very beneficial for the management and control programs of this disease.

## 1. Introduction

Brazil has the biggest western buffalo herd, where approximately 65% of which is located in the north region of this country [1]. Nowadays, the buffalo has been highlighted in the national scenery, showing more than just an alternative to the occupation of lands unsuitable for cattle but becoming an economically important option. As consequence, concern about sanitary management has increased considerably, because the clinical, pathological, and epidemiological studies are still poorly studied in Latin America.

Buffaloes, when compared to other domestic livestock, are generally a resistance animal [2]. This is particularly impressive because most of them, especially the water buffaloes, live in hot and humid regions that are willing to have several infectious agents [2]. Although the reason is not clear, the effect on buffaloes is often less deleterious than that on cattle.

Toxoplasmosis is a widespread zoonosis caused by the coccidian protozoan *Toxoplasma gondii* which can parasitize human beings and many warm-blooded animals, inducing abortions and neonatal mortality in small ruminants [3]. *Neospora caninum* is structurally and biologically similar to *Toxoplasma gondii* [4], causing abortion and neonatal mortality in cattle, sheep, goats, and horses in many countries [5]. *Anaplasma marginale, Babesia bovis,* and *Babesia bigemina* are important tick-borne agents of cattle worldwide [6]. Although many studies have been conducted worldwide on the prevalence of these important pathogens in animals, few studies have been conducted on water buffaloes in Latin America [7–9]. The present work aimed to assess the prevalence of *N. caninum, T. gondii, A. marginale, B. bovis,* and *B. bigemina* among water buffaloes in the north region of Brazil.

## 2. Materials and Methods

### 2.1. Serum Samples

Field samples of blood from water buffalo (*n* = 4796) were collected from different farms in eight provinces in Marajó island (Soure, Salvaterra, Muaná, Chaves, Ponta de Pedras, Cachoeira do Arari, and Santa Cruz do Arari) and five provinces in Continent (Belém, Castanhal, Paragominas, Abaetetuba, and Mojú) in the north region of Brazil in 2011. The minimum sample size was calculated by the following formula of the Pan American Zoonoses Center [10]:

(1) $$\begin{array}{c} N=\frac{p*\left(100-p\right)Z^{2}}{{\left(d*p/100\right)}^{2}}, \end{array}$$

where *N* is the number of samples; *p* is the expected prevalence; *Z* is the degree of confidence; *d* is the margin of error

The expected prevalence for selected bovine pathogens of buffaloes was estimated to be 7%. The confidence interval was 95%, and the margin of error was 5%. Thus, the minimum sample size was 3,650 animals, and a total of 4,796 animals were used in this study. Whole blood samples were collected from caudal or jugular vein of individual water buffaloes. For serum samples, blood samples without EDTA were incubated at room temperature and then centrifuged at 3000 rpm for 15 min; the sera were collected and then stored at −20°C until use.

### 2.2. ELISA for *B. bovis*, *B. bigemina*, *T. gondii*, *N. caninum*, and *A. marginale*

Briefly, 100 *μ*L of each antigen diluted in 0.05 M carbonate/bicarbonate buffer, pH 9.6, was added to each well of a micro-ELISA plate (Immulon; Dynatech Laboratories Inc.) and protein concentration was adjusted to 5 *μ*g/mL (*B. bovis*, *B. bigemina* and *T. gondii*) or 10 *μ*g/mL (*N. caninum,* and *A. marginale*). The plates were sealed and incubated overnight at 4°C. Plates were blocked for 1 h at 37°C in a humid chamber with 3% skim milk in carbonate/bicarbonate buffer. After five washes with buffer (phosphate buffered saline, pH 7.2, and 0.05% Tween 20, PBS-Tween), 100 *μ*L of diluted bovine sera (1 : 400) in PBS-Tween plus 5% normal rabbit serum was added in duplicate to the ELISA plate. Plates were incubated at 37°C in a humid chamber for 90 min and then washed five times with PBS-Tween. A 100 *μ*L aliquot of a 1 : 10000 dilution of alkaline phosphatase conjugated antibovine IgG (Sigma Chemical Co.) was added to each well and the plates were incubated at 37°C under the same conditions for 90 min. Plates were washed five times with PBS-Tween. The appropriate substrate (p-nitrophenyl phosphate) was added and the plates were sealed and incubated for 40 min at room temperature. The plates were then read at 405 nm wavelength on a micro-ELISA reader (B.T.-100; Embrabio, São Paulo, Brazil). The cutoff values were calculated based on 10 noninfected water buffaloes sera [11].

### 2.3. IFAT for *T. gondii*, *N. caninum*, *A. marginale*, *B. bovis*, and *B. bigemina*

Briefly, a 10 *μ*L field serum sample diluted in PBS (1 : 40) was applied as the first antibody on the fixed smears and then incubated for 1 h at 37°C in a moist chamber. After washing with PBS three times, the fluorescein isothiocyanate (FITC)-conjugated sheep antibovine IgG antibody (Sigma, St. Louis, MO, USA) was applied as a secondary antibody (1 : 300) and then incubated for 1 h at 37°C. The slides were washed three times with PBS and then examined using a fluorescent microscope (E400 Eclipse, Nikon, Kawasaki, Japan). Positive and negative control sera were added to each slide.

### 2.4. Statistical Analysis

The kappa coefficient was calculated to evaluate the agreement among the ELISA and IFAT. The chi-square test was used to evaluate significant differences (*P* < 0.05) of infection rate (*T. gondii, N. caninum, A. marginale, B. bovis,* and *B. bigemina*) in animals of different breed, reproductive status, and locations. The operational procedures were done using the R statistical software (R Foundation for Statistical Computing, version 2.12.2, 2011).

## 3. Results and Discussion

IgG antibodies of *T. gondii* were detected in 41.33% (1982/4796) and 35.77% (1715/4796) of sampled buffaloes, by ELISA and IFAT, respectively (Table 1).

Few reports have been done about the occurrence of *T. gondii* among buffaloes in Brazil [7]. Previous studies have reported a lower seroprevalence of this agent when compared to that observed among cattle. For instance, seroprevalence rates of 3.85%, 3.2%, and 1.1% were found among buffaloes in states of Bahia [3], São Paulo [12], and Pará [12], respectively. A lower seroprevalence of *T. gondii* among buffaloes has also been reported in Turkey [13], Egypt [14], Vietnam [15], and Iran [16].

Antibodies of *N. caninum* were detected in 55.55% (2665/4796) and 48.88% (2345/4796) of sampled buffaloes, by ELISA and IFAT, respectively (Table 1). Antibodies titers detected in seropositive animals ranged from 100 (cutoff) to 800: 100 (50%), 200 (25%), 400 (13.6%), and 800 (11.4%). Previous studies in the state of Pará have reported a seroprevalence of *N. caninum* ranging from 40.9% [17] to 70.15% [18] among buffaloes. On the other hand, a seroprevalence of 36.5% of *N. caninum* has been found among buffaloes from the state of Bahia [3]. The seroprevalence found here was higher than that found among buffaloes from Italy (34.6%) [19] but lower than that found in Egypt (68%) [14] and Argentina (64%) [20]. However, it is noteworthy to mention that differences in reported seroprevalence could be explained also by the test utilized.

Herein, 64.7% (3073/4796) and 50.0% (2399/4796) of sampled buffaloes showed IgG antibodies to *A. marginale*, by ELISA and IFAT, respectively (Table 1). Although previous studies reported the occurrence of *Babesia* sp. among buffaloes [9, 11], those regarding the prevalence of *A. marginale* are scarce. In Brazil, seroprevalence of *A. marginale* among buffaloes (ranging from 31.1 to 80%) is much different from that found among cattle (reaching in most cases 100%) [21, 22]. In the present study, sampled buffaloes did not show clinical signs of anaplasmosis. Although clinical anaplasmosis is not usually seen among *A. marginale* infected buffaloes, manifestation of diseases such as hemolytic anemia, inapetence, depression, emaciation, melena, tachycardia, tachypnea, constipation, jaundice, and pale mucous was reported in buffaloes from India showing a low parasitaemia [23].

IgG antibodies to *B. bovis* and *B. bigemina* were detected in 24.9% (1193/4796) and 22.76% (1092/4796) by ELISA and 22.5% (1079/4796) and 18.74% (899/4796) by IFAT of sampled buffaloes, respectively (Table 1). Thirteen percent (623/4796) of animals were seropositive to both *B. bovis* and *B. bigemina*.

The low seroprevalence of *B. bovis* and *B. bigemina* found in the present study suggests a low transmission rate in the studied area due to the habitat where buffaloes are maintained, characterized by woods isolated from cattle infested by *Babesia* sp. infected ticks. A Low exposure of buffaloes to babesiosis agents could be explained by the fact that those animals live submerged in muddy waters, diminishing the probability of attachment of ticks and consequently the transmission of studied pathogens [8, 11]. These findings classify the studied area as endemically instable for babesiosis agents in buffaloes, suggesting an elevated risk to babesiosis outbreaks in bovines, mainly in regions where cattle is raised closed to buffaloes or even when new animals are introduced in such herds.

The present study provides important information about the prevalence of *T. gondii, N. caninum, A. marginale, B. bovis,* and *B. bigemina* infections in water buffaloes. The real role of water buffaloes on the epidemiology of these diseases and, consequently, the impact of management and control programs targeting these animals should be determined.

## Figures and Tables

**Table 1:** Summary of the serological detection of *T. gondii, N. caninum, A. marginale, B. bovis*, and* B. bigemina* using ELISA and IFAT. The results of ELISA were cross-tabulated with those of IFAT.

Agent	ELISA^a^	IFAT^b^		ELISA/IFAT^c^
		(+)	(−)	
*Toxoplasma gondii *				
(+)	1934	1672	262	1936
(−)	2862	2	2860	2860
	**4796**	**1674**	**3122**	**4796**
*Neospora caninum *				
(+)	2599	2285	314	2601
(−)	2197	2	2195	2195
	**4796**	**2287**	**2509**	**4796**
*Anaplasma marginale *				
(+)	2997	2393	604	3002
(−)	1799	5	1794	1794
	**4796**	**2398**	**2398**	**4796**
*Babesia bigemina *				
(+)	1092	891	201	1100
(−)	3704	8	3696	3696
	**4796**	**899**	**3897**	**4796**
*Babesia bovis *				
(+)	1193	1075	118	1197
(−)	3603	4	3599	3599
	**4796**	**1079**	**3717**	**4796**

^a^Number of positive and negative buffaloes in ELISA assays.

^b^Number of positive and negative buffaloes in both ELISA and IFAT assays.

^c^The frequencies of positive and negative samples of combined ELISA and IFAT results.
